# Risk factors for COVID-19 diagnosis, hospitalization, and subsequent all-cause mortality in Sweden: a nationwide study

**DOI:** 10.1007/s10654-021-00732-w

**Published:** 2021-03-11

**Authors:** Jonathan Bergman, Marcel Ballin, Anna Nordström, Peter Nordström

**Affiliations:** 1 Unit of Geriatric Medicine, Department of Community Medicine and Rehabilitation, Umeå University, Umeå, Sweden; 2 Department of Public Health and Clinical Medicine, Section of Sustainable Health, Umeå University, Umeå, Sweden; 3 School of Sport Sciences, UiT the Arctic University of Norway, Tromsø, Norway

**Keywords:** Case–control study, Cohort study, Coronavirus, COVID-19, Epidemiology, SARS-CoV-2

## Abstract

We conducted a nationwide, registry-based study to investigate the importance of 34 potential risk factors for coronavirus disease 2019 (COVID-19) diagnosis, hospitalization (with or without intensive care unit [ICU] admission), and subsequent all-cause mortality. The study population comprised all COVID-19 cases confirmed in Sweden by mid-September 2020 (68,575 non-hospitalized, 2494 ICU hospitalized, and 13,589 non-ICU hospitalized) and 434,081 randomly sampled general-population controls. Older age was the strongest risk factor for hospitalization, although the odds of ICU hospitalization decreased after 60–69 years and, after controlling for other risk factors, the odds of non-ICU hospitalization showed no trend after 40–49 years. Residence in a long-term care facility was associated with non-ICU hospitalization. Male sex and the presence of at least one investigated comorbidity or prescription medication were associated with both ICU and non-ICU hospitalization. Three comorbidities associated with both ICU and non-ICU hospitalization were asthma, hypertension, and Down syndrome. History of cancer was not associated with COVID-19 hospitalization, but cancer in the past year was associated with non-ICU hospitalization, after controlling for other risk factors. Cardiovascular disease was weakly associated with non-ICU hospitalization for COVID-19, but not with ICU hospitalization, after adjustment for other risk factors. Excess mortality was observed in both hospitalized and non-hospitalized COVID-19 cases. These results confirm that severe COVID-19 is related to age, sex, and comorbidity in general. The study provides new evidence that hypertension, asthma, Down syndrome, and residence in a long-term care facility are associated with severe COVID-19.

**Supplementary Information:**

The online version contains supplementary material available at 10.1007/s10654-021-00732-w.

## Introduction

In December 2019, the Chinese city of Wuhan experienced an outbreak of a severe form of pneumonia of unknown cause. By January 7, the pathogen had been identified as a novel coronavirus, the severe acute respiratory syndrome coronavirus 2 (SARS-CoV-2) [1]. The disease was later designated coronavirus disease 2019 (COVID-19) by the World Health Organization [2]. Although a travel ban in and out of Wuhan was imposed [3], the virus spread around the globe, and COVID-19 was declared a global pandemic on March 11, 2020 [2]. Consequently, there is a large public-health need to study risk factors for developing severe COVID-19. Such studies can help inform strategies for vaccination and disease prevention.

Observational studies have shown that older age, male sex, and comorbidity in general are important risk factors for developing severe COVID-19 [4–8]. However, the results of these studies are inconsistent for particular comorbidities, such as hypertension [5–9], respiratory diseases [4, 6, 7], and cardiovascular disease [5, 7, 8]. Severe COVID-19 has also been associated with demographic factors such as minority ethnicity and low income [7]. The limitations of previous observational studies include small sample size [5, 6, 10], focus on particular risk factors [11, 12], lack of general-population control group [4–6, 8–10], and focus on a few particular outcomes, such as COVID-19-related death [7, 12], in-hospital mortality [4, 5, 9], or intensive care unit (ICU)/critical care admission [4, 5]. Therefore, we used data from Swedish national registries to investigate the importance of potential medical and demographic risk factors for COVID-19 diagnosis, hospitalization (with or without ICU admission), and subsequent all-cause mortality during the first wave of COVID-19.

## Methods

### Data

The Public Health Agency of Sweden provided data from its SmiNet database on all cases of COVID-19 confirmed in Sweden until mid-September 2020. Reporting confirmed cases to SmiNet is required by law. We did not have information on the methods of testing used to diagnose the COVID-19 cases. Only the first date of diagnosis or positive test for each individual was provided.

We obtained a control population by requesting that Statistics Sweden (the agency of government statistics) randomly sample 5 non-diagnosed individuals for each COVID-19 case. Each control was residing in Sweden on January 1, 2020, and was alive on January 31, 2020. No matching was performed. Statistics Sweden also provided registry data on sex, year and month of birth, country of birth, highest level of completed education in 2018, disposable family income in 2018, home municipality (on December 31, 2019), and a pseudo-anonymized household identifier, indicating which persons lived at the same street address (on December 31, 2019).

The Swedish National Board of Health and Welfare provided registry data on deaths, diagnoses, hospitalizations, prescription medication use, residence in long-term care facility, and use of homemaker service. All deaths in Sweden are recorded in the Cause of Death Register [13]. Diagnoses and hospitalizations are registered in the National Patient Register, to which all health care providers have been required to report hospitalizations since 1987 and physician visits in secondary care (i.e., non-primary care) since 2001 [14]. Each hospitalization or visit is assigned a main diagnosis and one or more secondary diagnoses, which indicate the purpose(s) of the hospitalization or visit. Medical and surgical procedures are also recorded. The National Patient Register has been validated, showing positive predictive values of more than 90% for most diagnoses, although sensitivity is often lower [14]. For cancer, we also selected diagnoses recorded in the Swedish Cancer Register from 1964 through 2018, so as to obtain a longer lookback period than is possible with the National Patient Register. Health-care providers have been required to report new cancer diagnoses to the Swedish Cancer Register since 1958 [15]. Data on prescription medication use were obtained from the Prescribed Drug Registry, which records all prescription medications collected at pharmacies in Sweden since July 2005 [16]. Data on residence in a long-term care facility and use of homemaker service were available from the Register for Care and Services for the Elderly and for Persons with Impairments According to the Social Services Act. Local governments are required to report to this register, whose quality is considered adequate for publication by the National Board of Health and Welfare [17]. Homemaker services are domestic services provided to persons (primarily older persons) who live at home but need help with shopping, cleaning, meal preparation, and similar tasks. Local governments are responsible for determining eligibility for these services, although the services may be provided by private businesses.

Information about ICU care was obtained from the Swedish Intensive Care Registry. In 2019, 83 of Sweden’s 84 ICUs reported to this registry [18]. The data from the registries were linked using the Personal Identification Number that is issued to each resident of Sweden at the time of birth or immigration. We obtained data files in which Personal Identification Numbers had been replaced by pseudo-anonymized identifiers, generated by Statistics Sweden. The study was approved by the Swedish Ethical Review Authority, who waived the requirement of obtaining informed consent (number 2020–02552).

### Variables

We investigated three different outcomes related to COVID-19 infection, each reflecting an increased severity of infection: COVID-19 diagnosis without hospitalization; non-ICU hospitalization with confirmed COVID-19 as the main diagnosis (International Classification of Diseases, 10^th^ Revision, Swedish Version, [ICD-10-SE] Code: U071); and ICU hospitalization for confirmed COVID-19 (ICD-10-SE: U071). ICU hospitalizations were traced in SmiNet and non-ICU hospitalizations were traced in the National Patient Register until October 1, 2020. These groups, along with the control group, were followed-up for all-cause mortality until October 1, 2020.

Table 1 shows the demographic variables, comorbidities, and prescription medications that were included in the analysis as potential risk factors for COVID-19. Although pneumonia is sometimes a complication of COVID-19, we included history of pneumonia as a potential risk factor because it may be a marker of frailty or impaired immune system. Variable definitions are available in Supplemental Tables 1 and 2. A composite variable was also constructed, which indicated the presence of at least one of the comorbidities or medications.

**Table 1:** Characteristics COVID-19 cases and controls

Variable	Controls (n = 434,081)	Diagnosis (n = 68,575)	Non-ICU hosp. (n = 13,589)	ICU hosp. (n = 2494)
Demographics				
Male sex, n (%)	218,736 (50.4)	26,808 (39.1)	7619 (56.1)	1814 (72.7)
Age, y				
Mean (SD)	41 (24)	46 (21)	65 (19)	59 (14)
0–19, n (%)	100,221 (23.1)	4,556 (6.6)	161 (1.2)	25 (1.0)
20–29, n (%)	54,657 (12.6)	13,128 (19.1)	381 (2.8)	91 (3.6)
30–39, n (%)	57,490 (13.2)	11,976 (17.5)	821 (6.0)	106 (4.3)
40–49, n (%)	54,872 (12.6)	12,002 (17.5)	1458 (10.7)	273 (10.9)
50–59, n (%)	54,808 (12.6)	11,896 (17.3)	2313 (17.0)	640 (25.7)
60–69, n (%)	47,074 (10.8)	5623 (8.2)	2137 (15.7)	753 (30.2)
70–79, n (%)	42,182 (9.7)	2946 (4.3)	2550 (18.8)	495 (19.8)
80–89, n (%)	18,653 (4.3)	3861 (5.6)	2743 (20.2)	107 (4.3)
≥ 90, n (%)	4,124 (1.0)	2587 (3.8)	1025 (7.5)	4 (0.2)
Born in Sweden, n (%)	349,128 (80.4)	51,281 (74.8)	8307 (61.1)	1376 (55.2)
Education in 2018, n (%)				
Missing^a^	100,490	3,709	767	125
Primary	66,656 (20.0)	11,837 (18.2)	4088 (31.9)	625 (26.4)
Secondary	144,543 (43.3)	27,705 (42.7)	5306 (41.4)	1075 (45.4)
Post-secondary, < 3 y	47,858 (14.3)	9011 (13.9)	1440 (11.2)	292 (12.3)
Post-secondary, ≥ 3 y	74,534 (22.3)	16,313 (25.1)	1988 (15.5)	377 (15.9)
Family disposable income in 2018, n (%)				
Missing^a^	88,321	2229	237	43
Quintile 1	68,766 (19.9)	12,120 (18.3)	4409 (33.0)	575 (23.5)
Quintile 2	69,086 (20.0)	12,407 (18.7)	2916 (21.8)	522 (21.3)
Quintile 3	69,225 (20.0)	11,500 (17.3)	2429 (18.2)	487 (19.9)
Quintile 4	69,343 (20.1)	14,386 (21.7)	1883 (14.1)	432 (17.6)
Quintile 5	69,340 (20.1)	15,933 (24.0)	1715 (12.8)	435 (17.7)
Long-term care facility, n (%)	3928 (0.9)	6121 (8.9)	1183 (8.7)	18 (0.7)
Homemaker service, n (%)	13,144 (3.0)	6825 (10.0)	4507 (33.2)	323 (13.0)
Stockholm residence^b^				
Missing	790	236	69	11
N (%)	100,008 (23.1)	16,400 (24.0)	5636 (41.7)	895 (36.0)
Comorbitities, n (%)				
Any comorbidity/medication	267,702 (61.7)	50,441 (73.6)	12,464 (91.7)	2218 (88.9)
Cardiovascular disease	31,922 (7.4)	6438 (9.4)	4433 (32.6)	445 (17.8)
Hypertension	94,582 (21.8)	16,416 (23.9)	8280 (60.9)	1340 (53.7)
Cancer	33,251 (7.7)	5515 (8.0)	2861 (21.1)	304 (12.2)
Immune disorder	1125 (0.3)	207 (0.3)	72 (0.5)	16 (0.6)
Autoimmune disease	19,372 (4.5)	3655 (5.3)	1551 (11.4)	189 (7.6)
Diabetes	26,026 (6.0)	4897 (7.1)	3,394 (25.0)	651 (26.1)
COPD	13,133 (3.0)	2168 (3.2)	1,578 (11.6)	169 (6.8)
Asthma	27,746 (6.4)	4493 (6.6)	1,208 (8.9)	211 (8.5)
Renal failure/chronic kidney disease	4720 (1.1)	1353 (2.0)	1,351 (9.9)	130 (5.2)
Glomerular disease	1569 (0.4)	337 (0.5)	213 (1.6)	42 (1.7)
Liver disease	2628 (0.6)	511 (0.7)	285 (2.1)	66 (2.6)
Dementia/Alzheimer’s disease	3665 (0.8)	3618 (5.3)	870 (6.4)	8 (0.3)
Down syndrome	297 (0.1)	57 (0.1)	20 (0.1)	8 (0.3)
HIV/AIDS	357 (0.1)	84 (0.1)	32 (0.2)	9 (0.4)
Sepsis	3448 (0.8)	1018 (1.5)	683 (5.0)	78 (3.1)
Influenza	3074 (0.7)	922 (1.3)	538 (4.0)	58 (2.3)
Pneumonia	17,422 (4.0)	3861 (5.6)	2193 (16.1)	234 (9.4)
Solid organ transplantation	408 (0.1)	85 (0.1)	110 (0.8)	25 (1.0)
Alcohol intoxication	11,754 (2.7)	2092 (3.1)	605 (4.5)	119 (4.8)
Prescription medications, n (%)				
Antithrombotics	71,744 (16.5)	13,797 (20.1)	6945 (51.1)	941 (37.7)
Lipid-modifying agents	58,383 (13.4)	9,168 (13.4)	5652 (41.6)	914 (36.6)
Proton-pump inhibitors	111,107 (25.6)	24,016 (35.0)	7726 (56.9)	1226 (49.2)
Corticosteroids, systemic	87,788 (20.2)	17,651 (25.7)	5543 (40.8)	879 (35.2)
Immunosuppressants	8724 (2.0)	1,614 (2.4)	782 (5.8)	137 (5.5)
Antivirals	32,695 (7.5)	7,624 (11.1)	1851 (13.6)	286 (11.5)
Opioids	137,169 (31.6)	29,214 (42.6)	8852 (65.1)	1410 (56.5)

*COPD* chronic obstructive pulmonary disease, *HIV/AIDS* human immunodeficiency virus/acquired immunodeficiency syndrome, *hosp.* hospitalization, *ICU* intensive care unit, *SD* standard deviation

^a^Part of the missing data on education and family income was due to a lack of data on persons aged < 15 years on Dec 31, 2018: 83,198 persons in the control group; 1,616 persons in the diagnosis-only group; 108 persons in the non-ICU hospitalized group; and 15 persons in the ICU hospitalized group

^b^Residence in Stockholm County (the capital of Sweden) refers to December 31, 2019

**Table 2:** Association between potential risk factors and COVID-19 (reference group: general population controls)

Variable	Unadjusted Odds Ratio (95% CI)			Adjusted^a^ Odds Ratio (95% CI)		
	Diagnosis	Non-ICU hosp.	ICU hosp.	Diagnosis	Non-ICU hosp.	ICU hosp.
Demographics						
Male sex	0.63 (0.62–0.64)	1.26 (1.21–1.30)	2.63 (2.41–2.87)	0.64 (0.63–0.65)	1.59 (1.53–1.65)	3.04 (2.76–3.35)
Age group, y						
0–19	1 (ref)	1 (ref)	1 (ref)	1 (ref)	1 (ref)	1 (ref)
20–29	5.28 (5.10–5.47)	4.34 (3.61–5.22)	6.67 (4.29–10.39)	0.69 (0.66–0.73)	2.45 (1.69–3.55)	3.62 (1.45–9.02)
30–39	4.58 (4.42–4.75)	8.89 (7.51–10.53)	7.39 (4.78–11.43)	0.47 (0.44–0.50)	4.12 (2.86–5.93)	3.70 (1.49–9.21)
40–49	4.81 (4.64–4.99)	16.54 (14.05–19.47)	19.94 (13.24–30.04)	0.46 (0.43–0.49)	7.25 (5.05–10.41)	9.68 (3.94–23.77)
50–59	4.77 (4.61–4.95)	26.27 (22.38–30.83)	46.81 (31.39–69.81)	0.45 (0.42–0.48)	10.17 (7.09–14.60)	20.30 (8.30–49.64)
60–69	2.63 (2.52–2.74)	28.26 (24.07–33.18)	64.12 (43.04–95.53)	0.24 (0.22–0.25)	8.46 (5.89–12.14)	23.97 (9.80–58.62)
70–79	1.54 (1.46–1.61)	37.63 (32.08–44.15)	47.04 (31.47–70.32)	0.10 (0.09–0.11)	7.31 (5.08–10.50)	14.59 (5.94–35.81)
80–89	4.55 (4.35–4.77)	91.54 (78.03–107.39)	23.00 (14.87- 35.55)	0.12 (0.11–0.13)	9.16 (6.36–13.19)	5.42 (2.17–13.57)
90	13.80 (13.03–14.61)	154.72 (130.66–183.22)	3.88 (1.35–11.17)	0.18 (0.16–0.19)	10.17 (7.02–14.75)	0.84 (0.22–3.19)
Born in Sweden	0.72 (0.71–0.74)	0.38 (0.37–0.40)	0.30 (0.28–0.32)	0.71 (0.69–0.72)	0.38 (0.37–0.40)	0.33 (0.30–0.37)
Education in 2018						
Primary	1 (ref)	1 (ref)	1 (ref)	1 (ref)	1 (ref)	1 (ref)
Secondary	1.08 (1.05–1.10)	0.60 (0.57–0.62)	0.79 (0.72–0.88)	1.11 (1.08–1.15)	0.87 (0.83–0.91)	0.91 (0.82–1.01)
Post-secondary, < 3 y	1.06 (1.03–1.09)	0.49 (0.46–0.52)	0.65 (0.57–0.75)	1.09 (1.05–1.13)	0.79 (0.74–0.85)	0.80 (0.69–0.92)
Post-secondary, ≥ 3 y	1.23 (1.20–1.26)	0.43 (0.41–0.46)	0.54 (0.47–0.61)	1.19 (1.15–1.23)	0.73 (0.69–0.78)	0.73 (0.63- 0.83)
Disposable family income in 2018						
Quintile 1	1 (ref)	1 (ref)	1 (ref)	1 (ref)	1 (ref)	1 (ref)
Quintile 2	1.02 (0.99–1.05)	0.66 (0.63–0.69)	0.90 (0.80–1.02)	1.30 (1.26–1.35)	0.98 (0.92–1.04)	0.93 (0.81–1.07)
Quintile 3	0.94 (0.92–0.97)	0.55 (0.52–0.58)	0.84 (0.75–0.95)	1.74 (1.69–1.80)	1.07 (1.00–1.14)	0.96 (0.83–1.10)
Quintile 4	1.18 (1.15–1.21)	0.42 (0.40–0.45)	0.75 (0.66–0.84)	1.88 (1.82–1.95)	0.99 (0.92–1.07)	0.94 (0.81–1.08)
Quintile 5	1.30 (1.27–1.34)	0.39 (0.36–0.41)	0.75 (0.66–0.85)	1.81 (1.75–1.88)	0.94 (0.87–1.01)	0.84 (0.72- 0.97)
Long-term care facility	10.73 (10.30–11.18)	10.44 (9.76–11.17)	0.80 (0.50–1.27)	9.96 (9.33–10.63)	1.17 (1.07–1.27)	0.39 (0.24- 0.63)
Homemaker service	3.54 (3.43–3.65)	15.89 (15.27–16.54)	4.76 (4.23–5.36)	2.24 (2.12–2.37)	5.30 (4.99–5.63)	4.53 (3.90–5.25)
Stockholm residence^b^	1.05 (1.03–1.07)	2.38 (2.30–2.47)	1.88 (1.73–2.04)	0.94 (0.92–0.96)	2.28 (2.19–2.37)	1.80 (1.64–1.96)
Comorbitities						
Any comorbidity/medication	1.73 (1.70–1.76)	6.89 (6.48–7.32)	4.99 (4.41–5.66)	1.31 (1.29–1.34)^c^	2.39 (2.23–2.57)^c^	2.17 (1.88–2.50)^c^
Cardiovascular disease	1.31 (1.27–1.34)	6.10 (5.88–6.34)	2.74 (2.47–3.03)	1.10 (1.05–1.15)	1.08 (1.02–1.14)	0.74 (0.65–0.85)
Hypertension	1.13 (1.11–1.15)	5.60 (5.40–5.80)	4.17 (3.85–4.51)	1.00 (0.97–1.03)	1.24 (1.18–1.31)	1.42 (1.27–1.58)
Cancer	1.05 (1.02–1.09)	3.21 (3.08–3.36)	1.67 (1.48–1.89)	0.97 (0.93–1.00)	1.01 (0.96–1.07)	0.81 (0.71–0.93)
Immune disorder	1.17 (1.00–1.35)	2.05 (1.61–2.60)	2.48 (1.51–4.08)	1.01 (0.86–1.19)	1.33 (1.01–1.73)	1.79 (1.04–3.09)
Autoimmune disease	1.21 (1.16–1.25)	2.76 (2.61–2.91)	1.76 (1.51–2.04)	0.97 (0.93–1.02)	1.13 (1.05–1.21)	0.95 (0.80–1.14)
Diabetes	1.21 (1.17–1.24)	5.22 (5.01–5.44)	5.54 (5.07–6.07)	1.13 (1.09–1.18)	1.54 (1.46–1.62)	1.82 (1.62–2.03)
COPD	1.05 (1.00–1.10)	4.21 (3.98–4.45)	2.33 (1.99–2.73)	1.07 (1.01–1.13)	1.37 (1.28–1.47)	1.12 (0.94–1.34)
Asthma	1.03 (0.99–1.06)	1.43 (1.35–1.52)	1.35 (1.17–1.56)	1.02 (0.98–1.06)	1.22 (1.13–1.31)	1.53 (1.30–1.79)
Renal failure/chronic kidney disease	1.83 (1.72–1.95)	10.04 (9.43–10.70)	5.00 (4.18–5.98)	1.29 (1.19–1.39)	1.47 (1.36–1.60)	1.18 (0.95–1.46)
Glomerular disease	1.35 (1.20–1.52)	4.17 (3.60–4.83)	4.33 (3.14–5.97)	1.03 (0.90–1.17)	1.20 (1.00–1.43)	1.40 (0.97–2.02)
Liver disease	1.23 (1.12–1.36)	3.52 (3.11–3.98)	4.46 (3.48–5.72)	0.99 (0.90–1.10)	1.07 (0.93–1.23)	1.37 (1.05–1.79)
Dementia/Alzheimer’s disease	6.54 (6.24–6.85)	8.03 (7.45–8.67)	0.38 (0.19–0.76)	1.92 (1.79–2.05)	1.09 (0.99–1.20)	0.15 (0.07–0.31)
Down syndrome	1.21 (0.91–1.61)	2.16 (1.37–3.40)	4.52 (2.21–9.25)	1.77 (1.04–2.99)	3.24 (1.55–6.78)	4.26 (1.01–17.90)
HIV/AIDS	1.50 (1.18–1.90)	3.17 (2.24–4.49)	4.87 (2.58–9.18)	0.99 (0.77–1.28)	1.13 (0.76–1.68)	1.45 (0.73–2.89)
Sepsis	1.88 (1.75–2.02)	6.61 (6.08–7.19)	4.23 (3.38–5.28)	1.25 (1.15–1.36)	1.20 (1.08–1.33)	1.23 (0.96–1.59)
Influenza	1.92 (1.78–2.06)	5.76 (5.24–6.32)	3.47 (2.68–4.49)	1.23 (1.13–1.34)	1.47 (1.31–1.64)	1.36 (1.02–1.80)
Pneumonia	1.43 (1.38–1.48)	4.60 (4.39–4.83)	2.47 (2.16–2.83)	1.12 (1.07–1.17)	1.44 (1.35–1.53)	1.22 (1.05–1.43)
Solid organ transplantation	1.32 (1.04–1.67)	8.68 (7.02–10.72)	10.76 (7.17–16.15)	0.92 (0.71–1.19)	1.41 (1.07–1.84)	1.36 (0.82–2.26)
Alcohol intoxication	1.13 (1.08–1.19)	1.68 (1.54–1.82)	1.80 (1.50–2.17)	0.86 (0.82–0.90)	0.76 (0.69–0.84)	0.80 (0.66- 0.97)
Prescription medications						
Antithrombotics	1.27 (1.25–1.30)	5.28 (5.10–5.47)	3.06 (2.82–3.32)	1.01 (0.98–1.04)	1.19 (1.13–1.25)	1.18 (1.06–1.32)
Lipid-modifying agents	0.99 (0.97–1.02)	4.59 (4.43–4.75)	3.72 (3.43–4.04)	0.95 (0.91–0.98)	0.98 (0.93–1.03)	0.98 (0.87–1.09)
Proton-pump inhibitors	1.57 (1.54–1.59)	3.83 (3.70–3.97)	2.81 (2.60–3.04)	1.16 (1.14–1.19)	1.32 (1.27–1.38)	1.22 (1.11–1.33)
Corticosteroids, systemic	1.37 (1.34–1.39)	2.72 (2.62–2.81)	2.14 (1.97–2.33)	1.11 (1.09–1.14)	1.26 (1.21–1.32)	1.27 (1.16–1.40)
Immunosuppressants	1.18 (1.11–1.24)	2.98 (2.76–3.21)	2.83 (2.38–3.37)	0.90 (0.85–0.97)	1.08 (0.98–1.20)	1.22 (0.97–1.54)
Antivirals	1.54 (1.50–1.58)	1.94 (1.84–2.04)	1.59 (1.40–1.80)	1.17 (1.14–1.20)	1.04 (0.99–1.11)	1.04 (0.91–1.19)
Opioids	1.61 (1.58–1.63)	4.05 (3.90–4.19)	2.82 (2.60–3.05)	1.16 (1.14–1.18)	1.34 (1.28–1.40)	1.16 (1.05–1.27)

*COPD* chronic obstructive pulmonary disease, *HIV/AIDS* human immunodeficiency virus/acquired immunodeficiency syndrome, *hosp.* hospitalization, *ICU* intensive care unit

^a^Adjusted for all variables in column 1 except for Any Comorbidity/Medication

^b^Residence in Stockholm County (the capital of Sweden) refers to December 31, 2019

^c^Adjusted for all demographic variables

### Statistical analysis

Multinomial logistic regression was used to estimate odds ratios for COVID-19 diagnosis or ICU or non-ICU hospitalization. Cox regression was used to estimate hazard ratios for mortality. In each model, persons were excluded if they had missing data on at least one of the included variables.

For non-hospitalized COVID-19 cases, baseline date was defined as the date of testing or diagnosis, whichever came first or was available. If both dates were unavailable, the case was excluded from the analysis. Baseline date for hospitalized cases was the date of ICU or non-ICU admission. For controls, we randomly sampled a date from the baseline dates among cases. Controls who had died by their sampled date were excluded from the analysis.

A subgroup analysis was conducted to examine the prevalence of risk factors for COVID-19 and subsequent mortality in the age group 0–19 years. We did not estimate odds ratios and hazard ratios in this age group because of the small number of deaths and hospitalized COVID-19 cases.

The incidence of COVID-19 was estimated by dividing the number of cases by the size of the Swedish population (in total and by age group and sex) on December 31, 2019 [19]. The Kaplan–Meier method was used to estimate mortality rates, with confidence intervals based on the *log(survival)* transformation. All statistical analyses were performed in RStudio (R version 3.6.2). Statistical significance was determined by 95% confidence intervals not overlapping 1.

## Results

There were 87,069 confirmed cases of COVID-19 (incidence rate in Swedish population: 843 cases/100,000 population) and 435,345 general-population controls. Of the cases, 84,633 (97.2%) had an available date of testing and/or diagnosis, which ranged from January 30, 2020, to September 27, 2020. Most cases were diagnosed between April and June (Supplemental Fig. 1). The incidence was higher in women than in men in most age groups (Supplemental Fig. 2). The incidence was lowest in the age group 0–19 years and highest in the age group ≥ 90 years.

**Fig. 1:**
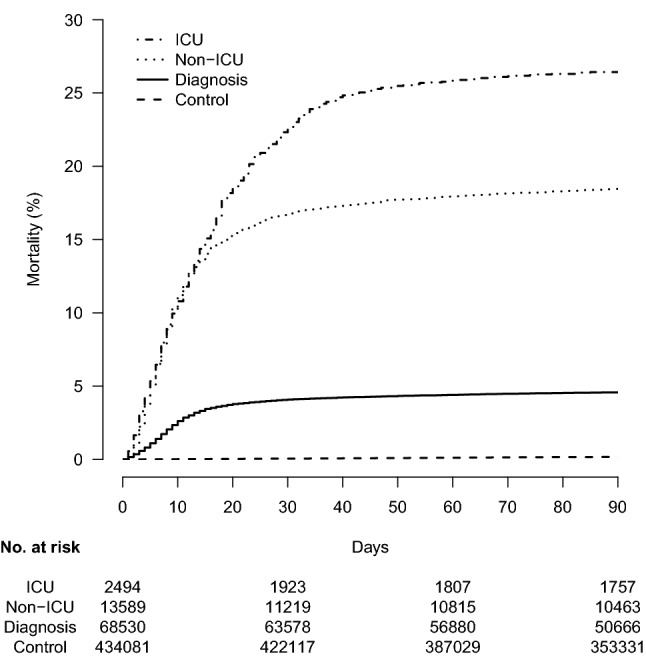
Risk of all-cause mortality in Swedish COVID-19 cases (intensive care unit [ICU] hospitalized, non-ICU hospitalized, and diagnosed only) confirmed by mid-September 2020 and in general-population controls

By October 1, 2020, 13,589 (15.6%) of the COVID-19 cases had been non-ICU hospitalized for confirmed COVID-19 and 2,494 (2.9%) had been ICU hospitalized. The 16,083 hospitalized patients lived in 14,936 different households (479 [3.0%] patients had missing data on household). Of these households, 95.7% (n = 14,287) had only one hospitalized household member (mean 1.04 hospitalized persons per household).

### Risk factors for COVID-19 diagnosis and hospitalization

In the analysis of risk factors for COVID-19 diagnosis and hospitalization, 1264 controls were excluded because they died prior to their assigned baseline date. Furthermore, 2411 non-hospitalized COVID-19 cases were excluded because no date of testing or diagnosis was available.

Risk factors for COVID-19 diagnosis and hospitalization are presented in Tables 1 and 2. The majority of non-hospitalized COVID-19 cases were women, but the majority of hospitalized cases were men. After adjustment for other risk factors, the odds of non-ICU hospitalization increased until the age group 40–49 years, after which there was no observable trend. Both the adjusted and unadjusted odds of ICU admission increased until the age group 60–69 years and decreased thereafter.

Being born in Sweden was associated with lower odds of COVID-19 diagnosis and hospitalization (Table 2). Higher levels of education were associated with higher odds of COVID-19 diagnosis but with lower odds of hospitalization. After adjustment for other risk factors, family disposable income was positively associated with COVID-19 diagnosis, but there was no clear association with hospitalization.

After adjustment for other risk factors, residence in a long-term care facility was associated with increased odds of diagnosis and, albeit to a lesser degree, non-ICU hospitalization (Table 2). Residence in a long-term care facility was associated with lower odds of ICU admission. Use of homemaker service was associated with both COVID-19 diagnosis and hospitalization (ICU and non-ICU).

Approximately 90% of ICU and non-ICU hospitalized patients had at least one of the investigated comorbidities or medications, and this was associated with more than twice the odds of ICU and non-ICU hospitalization after adjustment for demographic factors (Tables 1 and 2). The two comorbidities most strongly associated with ICU and non-ICU hospitalization after adjustment for other risk factors were diabetes and Down syndrome. Other comorbidities associated with both ICU and non-ICU hospitalization were hypertension, immune disorder, asthma, influenza, and pneumonia. Comorbidities significantly associated with increased odds of either ICU or non-ICU admission, but not with both, were autoimmune disease, chronic obstructive pulmonary disease (COPD), renal failure/chronic kidney disease, sepsis, solid organ transplantation, and liver disease. Human immunodeficiency virus/acquired immunodeficiency syndrome (HIV/AIDS) and glomerular disease were non-significantly associated with both ICU and non-ICU hospitalization. Alcohol intoxication was associated with lower odds of COVID-19 diagnosis and hospitalization after adjustment for other risk factors.

Cancer was not associated with increased odds of hospitalization after adjustment for other risk factors (Table 2). The unadjusted association was explained by sex, age group, hypertension, cardiovascular disease, corticosteroid use, and opioid use (Supplemental Table 3). Cancer in the past year, however, was associated with COVID-19 diagnosis and non-ICU hospitalization, but not with ICU hospitalization, after adjustment for all variables in Table 2 other than Any Comorbidity/Medication (adjusted odds ratio for diagnosis 1.17, 1.09–1.26; adjusted odds ratio for non-ICU hospitalization 1.30, 95% CI 1.20–1.42; adjusted odds ratio for ICU admission 0.90, 95% CI 0.72–1.12).

**Table 3:** All-cause mortality in COVID-19 cases and general population controls

Variable	Control (n = 434,081)	Diagnosis (n = 68,530)	Non-ICU hosp. (n = 13,589)	ICU hosp. (n = 2,494)
Number of deaths				
30 days	248	2792	2279	565
60 days	483	2999	2436	643
90 days	706	3098	2501	657
Total	1059	3286	2612	668
Person-months at risk	1,737,135	245,211	54,671	9516
Mortality rate/1,000 person-months	0.6	13.4	47.8	70.2
Mortality, % (95% CI)				
30 days	0.1 (0.1–0.1)	4.1 (3.9–4.2)	16.8 (16.2–17.4)	22.7 (21.0–24.3)
60 days	0.1 (0.1–0.1)	4.4 (4.3–4.6)	18.0 (17.3–18.6)	25.8 (24.1–27.6)
90 days	0.2 (0.2–0.2)	4.6 (4.4–4.7)	18.5 (17.8–19.1)	26.4 (24.7–28.1)

*hosp.* hospitalization, *ICU* intensive care unit

Cardiovascular disease was associated with slightly increased odds of non-ICU hospitalization, but with lower odds of ICU admission, after adjustment for other risk factors (Table 2). The unadjusted association of cardiovascular disease with ICU admission was explained by age group, sex, hypertension, and diabetes (Supplemental Table 4).

**Table 4:** Potential risk factors for all-cause mortality

Variable	Hazard Ratio (95% CI)		
	Unadjusted	Adjusted for age group, sex, and COVID-19	Fully adjusted^a^
COVID-19			
Control	1 (ref)	1 (ref)	1 (ref)
Diagnosis only	20.54 (19.17–22.02)	18.98 (17.67–20.37)	10.03 (9.27–10.86)
Non-ICU hospitalization	84.36 (78.54–90.61)	26.38 (24.54–28.36)	17.34 (16.04–18.75)
ICU hospitalization	120.26 (109.15–132.49)	92.92 (84.17–102.58)	77.41 (69.83–85.80)
Demographics			
Male sex	1.14 (1.09–1.19)	1.56 (1.49–1.64)	1.67 (1.59–1.76)
Age group, y			
0–19	1 (ref)	1 (ref)	1 (ref)
20–29	3.09 (1.32–7.23)	1.23 (0.53–2.88)	0.52 (0.17–1.62)
30–39	3.92 (1.74–8.84)	1.62 (0.72–3.66)	0.62 (0.21–1.88)
40–49	12.98 (6.24–27.01)	4.74 (2.28–9.86)	2.18 (0.79–6.00)
50–59	42.29 (20.89–85.59)	13.27 (6.55–26.88)	5.33 (1.98–14.37)
60–69	133.04 (66.21–267.34)	47.23 (23.49–94.96)	13.70 (5.10–36.76)
70–79	456.41 (227.87–914.17)	201.51 (100.55–403.84)	36.27 (13.53–97.23)
80–89	1683.57 (841.20–3369.51)	541.32 (270.30–1084.07)	67.65 (25.23–181.44)
≥ 90	3819.71 (1907.57–7648.56)	846.33 (422.31–1696.08)	93.13 (34.69–249.97)
Born in Sweden	1.02 (0.97–1.08)	0.96 (0.90–1.01)	1.00 (0.94–1.07)
Education in 2018			
Primary	1 (ref)	1 (ref)	1 (ref)
Secondary	0.42 (0.40–0.44)	0.93 (0.89–0.98)	0.96 (0.92–1.02)
Post-secondary, < 3 y	0.27 (0.25–0.29)	0.83 (0.76–0.90)	0.89 (0.81–0.97)
Post-secondary, ≥ 3 y	0.21 (0.20–0.23)	0.72 (0.67–0.78)	0.83 (0.77–0.91)
Disposable family income in 2018			
Quintile 1	1 (ref)	1 (ref)	1 (ref)
Quintile 2	0.43 (0.41–0.46)	0.78 (0.74–0.83)	0.88 (0.83–0.93)
Quintile 3	0.24 (0.22–0.26)	0.67 (0.62–0.72)	0.79 (0.73–0.85)
Quintile 4	0.11 (0.10–0.12)	0.57 (0.52–0.64)	0.71 (0.64–0.79)
Quintile 5	0.09 (0.08–0.10)	0.54 (0.49–0.61)	0.67 (0.59–0.74)
Long-term care facility	42.64 (40.76–44.61)	2.30 (2.17–2.44)	1.74 (1.63–1.85)
Homemaker service	42.55 (40.58–44.62)	1.91 (1.80–2.03)	1.41 (1.32–1.49)
Stockholm residence^b^	1.91 (1.82–2.00)	1.18 (1.12–1.23)	1.09 (1.04–1.15)
Comorbitities			
Any comorbidity/medication	43.72 (35.79–53.42)	2.57 (2.09–3.16)	2.34 (1.88–2.91)^c^
Cardiovascular disease	14.85 (14.19–15.54)	1.44 (1.37–1.51)	1.13 (1.07–1.20)
Hypertension	19.07 (17.91–20.31)	1.58 (1.47–1.69)	1.22 (1.14–1.32)
Cancer	6.47 (6.17–6.78)	1.16 (1.11–1.22)	1.14 (1.08–1.19)
Immune disorder	1.78 (1.29–2.46)	1.32 (0.96–1.82)	1.07 (0.77–1.48)
Autoimmune disease	3.37 (3.16–3.59)	1.24 (1.16–1.32)	1.08 (1.00–1.16)
Diabetes	5.86 (5.58–6.16)	1.39 (1.32–1.46)	1.23 (1.16–1.30)
COPD	5.53 (5.20–5.89)	1.35 (1.27–1.44)	1.08 (1.01–1.16)
Asthma	1.22 (1.12–1.33)	1.04 (0.95–1.13)	0.85 (0.78–0.93)
Renal failure/chronic kidney disease	14.83 (13.96–15.74)	1.69 (1.59–1.79)	1.30 (1.22–1.39)
Glomerular disease	3.28 (2.70–3.99)	1.48 (1.22–1.81)	0.87 (0.70–1.07)
Liver disease	4.10 (3.57–4.72)	1.74 (1.51–2.00)	1.27 (1.09–1.46)
Dementia/Alzheimer’s disease	28.29 (26.92–29.73)	1.90 (1.80–2.01)	1.60 (1.50–1.70)
Down syndrome	2.70 (1.62–4.47)	15.15 (9.09–25.24)	10.91 (5.41–22.02)
HIV/AIDS	0.85 (0.38–1.89)	1.24 (0.56–2.77)	0.80 (0.33–1.94)
Sepsis	10.10 (9.33–10.93)	1.51 (1.39–1.63)	1.12 (1.03–1.22)
Influenza	6.28 (5.67–6.95)	1.26 (1.14–1.39)	0.93 (0.84–1.04)
Pneumonia	7.57 (7.20–7.97)	1.61 (1.53–1.70)	1.30 (1.23–1.38)
Solid organ transplantation	5.03 (3.75–6.74)	3.02 (2.25–4.05)	1.66 (1.19–2.32)
Alcohol intoxication	2.12 (1.93–2.34)	1.55 (1.40–1.71)	1.12 (1.02–1.24)
Prescription medications			
Antithrombotics	15.14 (14.36–15.97)	1.50 (1.41–1.59)	1.16 (1.09–1.25)
Lipid-modifying agents	7.58 (7.25–7.93)	1.15 (1.10–1.21)	0.93 (0.88–0.98)
Proton-pump inhibitors	4.36 (4.16–4.57)	1.25 (1.20–1.31)	1.03 (0.98–1.08)
Corticosteroids, systemic	2.95 (2.82–3.08)	1.20 (1.15–1.26)	1.11 (1.05–1.17)
Immunosuppressants	2.51 (2.27–2.77)	1.26 (1.14–1.39)	1.00 (0.89–1.13)
Antivirals	2.00 (1.88–2.13)	1.04 (0.97–1.10)	0.98 (0.92–1.05)
Opioids	5.80 (5.51–6.10)	1.35 (1.28–1.42)	1.16 (1.09–1.22)

*COPD* chronic obstructive pulmonary disease, *HIV/AIDS* human immunodeficiency virus/acquired immunodeficiency syndrome, *hosp.* hospitalization, *ICU* intensive care unit

^a^Adjusted for all variables in column 1 except for Any Comorbidity/Medication

^b^On December 31, 2019

^c^Adjusted for the demographic variables

Antithrombotics, proton-pump inhibitors, corticosteroids, and opioids were associated with both ICU and non-ICU hospitalization for COVID-19 after adjustment other risk factors (Table 2). Antivirals and lipid-modifying agents were not associated with either ICU or non-ICU hospitalization after controlling for other risk factors. The unadjusted association for lipid-modifying agents was explained by age, sex, hypertension, diabetes, and cardiovascular disease (Supplemental Table 5). Immunosuppressants were associated with increased odds of ICU hospitalization and slightly increased odds of non-ICU hospitalization, although the associations were not significant.

### All-cause mortality in COVID-19

In the analysis of all-cause mortality, an additional 45 non-hospitalized COVID-19 cases were excluded because they died prior to the date of testing or diagnosis. The median number of days from baseline until the end date of the analysis (October 1, 2020) was 119 days (interquartile range, 98–155 days).

Excess mortality was observed in hospitalized and non-hospitalized COVID-19 cases after adjustment for other risk factors (Fig. 1, Table 3, Table 4). The only demographic factor not associated with all-cause mortality was birth in Sweden (Table 4). Most comorbidities were associated with mortality in excess of any risk conferred by COVID-19 (Table 4).

### COVID-19 and subsequent mortality in persons aged 0–19 years

Male sex was not associated with COVID-19 diagnosis or ICU or non-ICU hospitalization in persons aged 0–19 years (Supplemental Table 6). Hospitalized COVID-19 cases were less often born in Sweden, and they more often had hypertension, cancer, diabetes, COPD, sepsis, pneumonia, and corticosteroid use than did general-population controls and non-hospitalized COVID-19 cases. Asthma and proton-pump inhibitors were more common in non-ICU hospitalized and diagnosis-only COVID-19 cases than in general-population controls. There were too few deaths to evaluate excess mortality among COVID-19 cases aged 0–19 years (Supplemental Table 7).

## Discussion

This nationwide study confirmed that older age, male sex, and comorbidity in general are risk factors for COVID-19 hospitalization and, with the exception of male sex, risk factors for COVID-19 diagnosis without hospitalization. Older age was the strongest risk factor for COVID-19 hospitalization, but the odds of non-ICU hospitalization showed no trend after 40–49 years after adjustment for other risk factors, and the odds of ICU admission decreased after 60–69 years (with or without adjustment). Furthermore, persons living in long-term care facilities were rarely ICU admitted. Excess all-cause mortality was observed in both hospitalized and non-hospitalized cases, even after controlling for a large number of comorbidities and demographic factors. Children and young people aged 0–19 years were rarely diagnosed with or hospitalized for COVID-19, and there were too few deaths in this age group to evaluate excess mortality.

Almost all (90%) hospitalized patients had one of the investigated comorbidities or medications, and this was associated with more than twice the odds of ICU and non-ICU hospitalization for COVID-19. As in previous studies [5–7], age was the strongest risk factor for severe COVID-19, and none of the comorbidities or medications stood out as a particularly important risk factor for COVID-19 diagnosis or hospitalization. However, Down syndrome, although uncommon, was the condition most strongly associated with COVID-19 hospitalization after adjustment for other risk factors. Down syndrome was also associated with increased all-cause mortality. Previous data on Down syndrome as a risk factor for COVID-19 are limited but consistent with this result [20].

Our study also showed that both diabetes and hypertension were highly prevalent and positively associated with both ICU and non-ICU hospitalization for COVID-19. In previous studies, diabetes has often [5–7, 12], but not always [8, 9], been significantly associated with severe COVID-19 or COVID-19 mortality after controlling for other risk factors. Previous results for hypertension have been more contradictory [5–9]. We have no explanation for this. We defined hypertension as either a diagnosis of hypertension or a history antihypertensive treatment, so as to increase the sensitivity of detecting hypertension in the study population. Some antihypertensive treatments, namely angiotensin-converting enzyme inhibitors and angiotensin II receptor blockers, have been suspected to increase the risk of contracting COVID-19 and of developing severe COVID-19. However, three previous observational studies found no association with COVID-19 diagnosis [11, 21, 22], and only one of these found an association with severe COVID-19 [22]. Two clinical trials also showed that continuation of antihypertensive treatment did not lead to worse outcomes in hospitalized COVID-19 cases [23, 24]. Therefore, it is unlikely that the association with COVID-19 hospitalization in our study is due to an effect of the antihypertensive medications.

After adjustment for other risk factors, we found only a weak association between cardiovascular disease and non-ICU hospitalization for COVID-19, and we found no association with ICU hospitalization. This result contradicts the findings of systematic reviews and meta-analyses [25–27]. Previous observational studies have shown conflicting results, perhaps in part due to differences in definition [5, 7, 8]. Our results suggest that the association between cardiovascular disease and COVID-19 hospitalization is primarily driven by age, sex, hypertension, and diabetes.

We found that cancer in the past year, but not ever history of cancer, was associated with COVID-19 diagnosis and hospitalization. This finding corroborates the results of three previous observational studies and a meta-analysis [6–8, 27]. Altogether, only recent cancer appears to be associated with an increased risk of developing severe COVID-19, which could be due to the underlying treatment (e.g., chemotherapy and other medications that affect the immune system).

Our study showed that both COPD and asthma were associated with COVID-19 hospitalization, but only COPD was associated with all-cause mortality after adjustment for COVID-19, age group, and sex. Previous studies have shown conflicting results concerning the association between asthma and COPD and severe COVID-19 or mortality [4, 6, 7]. It is possible that the positive association in our study applies only to severe asthma, as we only had data on diagnoses made in secondary care and hospitalized care.

Concerning demographic factors, the burden of COVID-19 was greater in foreign-born persons, as they were more often diagnosed and hospitalized, although all-cause mortality was not higher in foreign-born persons. There was not a clear association between income and COVID-19 hospitalization, although having a disposable family income in the 3^rd^ to 5^th^ quintiles was associated with higher odds of COVID-19 diagnosis without hospitalization. Persons with higher levels of education were more often diagnosed and less often hospitalized with COVID-19. This association with COVID-19 diagnosis may at least in part be caused by more frequent testing of health care professionals than of the general population. Residence in a long-term care facility was a strong risk factor for COVID-19 diagnosis and subsequent all-cause mortality, although the association with non-ICU hospitalization was weaker and residents of long-term care facilities were rarely ICU admitted. It is well-known that long-term care facilities for older people have been severely affected by COVID-19 [28], but data on such residence has not been available in several large epidemiologic studies [4, 6–8]. Our results confirm that it is important to keep COVID-19 out of long-term care facilities.

A number limitations of this study need to be mentioned. First, the associations presented in this study are not necessarily causal, as there may be residual or unmeasured confounding. Second, the presence of some medical conditions, especially mild conditions, may be underestimated because primary-care diagnoses and complete histories of medical conditions were not available. Third, the likelihood that a person tests positive for COVID-19 is related not only to his or her risk of being infected, but also to his or her access to testing, willingness to be tested, and severity of symptoms. The Swedish strategy for testing was initially to prioritize hospitalized patients, health care professionals, and residents of long-term care facilities [29]. In April 2020, the strategy was extended to include personnel in essential sectors other than health care [30], and in June 2020, the strategy was extended to all persons with symptoms [31]. This fact may have biased our results for non-hospitalized COVID-19 cases, especially because most cases were diagnosed between April and June. However, the testing strategy should not have affected our results for hospitalized cases. Fourth, data on smoking and body mass index were not available. The strengths of the study include its nationwide coverage and the availability of many previously suspected risk factors for severe COVID-19. These strengths increase the internal and external validity of the results.

In summary, this nationwide study confirmed that severe COVID-19 is related to age, sex, and general health rather than particular medical conditions. As previously shown, diabetes was a strong risk factor, and only recent cancer was associated with severe COVID-19. Cardiovascular disease was weakly associated with COVID-19 hospitalization after controlling for other risk factors. This study provides new evidence that hypertension, asthma, Down syndrome, and residence in a long-term care facility are associated with severe COVID-19. Excess all-cause mortality was observed in both hospitalized and non-hospitalized COVID-19 cases.

## Supplementary Information

Below is the link to the electronic supplementary material.

Supplementary file1 (DOCX 153 KB)

## Data Availability

Individual-level data will not be shared because these are classified as secret under the Swedish Public Access to Information and Secrecy Act.

## Declarations

### Code availability

Statistical code may be obtained from JB upon request.
