# Supplementary material for: Risk factors for COVID-19 diagnosis, hospitalization, and subsequent all-cause mortality in Sweden: a nationwide study
Source: Eur J Epidemiol. 2021 Mar 11;36(3):287–98. doi: 10.1007/s10654-021-00732-w (PMC7946619; doi:10.1007/s10654-021-00732-w)
Supplement: Supplementary file 1 — Supplementary file1 (DOCX 153 KB) [file 10654_2021_732_MOESM1_ESM.docx]

**Supplementary Information**

**Title:** Risk Factors for COVID-19 Diagnosis, Hospitalization, and Subsequent All-Cause Mortality in Sweden: A Nationwide Study

**Journal Name:** European Journal of Epidemiology

**Author Names and Affiliations:** Jonathan Bergman (ORCID: 0000-0003-1904-6140),^1^ Marcel Ballin (ORCID: 0000-0002-9638-7208),^1,2^ Anna Nordström (ORCID: 0000-0003-3534-456X),^2,3^ Peter Nordström (ORCID: 0000-0003-2924-508X),^1^*

^1^Department of Community Medicine and Rehabilitation, Unit of Geriatric Medicine, Umeå University, Umeå, Sweden

^2^Department of Public Health and Clinical Medicine, Section of Sustainable Health, Umeå University, Umeå, Sweden

^3^School of Sport Sciences, UiT the Arctic University of Norway, Tromsø, Norway

***Corresponding author:**

Peter Nordström, Professor

Unit of Geriatric Medicine

Department of Community Medicine and Rehabilitation

Umeå University

90187 Umeå

Sweden

Phone: +4670 899 65 99

E-mail: peter.nordstrom@umu.se

**Supplemental Table 1** Definitions of comorbidities and prescription medications

**Supplemental Table 2** Definitions of demographic variables

**Supplemental Fig. 1** Number of COVID-19 cases (7-day moving average) in Sweden from March to September 2020

**Supplemental Fig. 2** Incidence of COVID-19 in Sweden until September 2020 by age group and sex

**Supplemental Table 3** Association between potential risk factors and COVID-19 (reference group: general population controls)

**Supplemental Table 4** Association between potential risk factors and COVID-19 (reference group: general population controls)

**Supplemental Table 5** Association between potential risk factors and COVID-19 (reference group: general population controls)

**Supplemental Table 6** Characteristics of COVID-19 cases and general-population controls aged 0-19 years

**Supplemental Table 7** All-cause mortality in COVID-19 cases and general-population controls aged 0-19 years

**Supplemental Table 1** Definitions of comorbidities and prescription medications

| **Variable** | **Definition** | **Code Type** | **Codes** |
| --- | --- | --- | --- |
| **Comorbidities** |  |  |  |
| Cardiovascular disease | Myocardial infarction | ICD-9/10-SE | I21, I22, 410 |
|  | Stroke | ICD-9/10-SE | I60-I64, 431-434 |
|  | Angina pectoris | ICD-9/10-SE | I20, 413 |
|  | Heart failure | ICD-9/10-SE | I50, 428 |
|  | Atrial fibrillation/flutter | ICD-9/10-SE | I48, 427D |
| Hypertension | Hypertension | ICD-10-SE | I10, I15 |
|  | Angiotensin-converting enzyme inhibitors/angiotensin II receptor blocker | ATC | C09 |
|  | Calcium-receptor blocker | ATC | C08 |
|  | Diuretic | ATC | C03 |
| Cancer^a^ | Malignant neoplasm | ICD-7/10-SE | C, 140-209 |
| Immune disorder | Immunodeficiency | ICD-10-SE | D80-D84 |
|  | Immunoglobulins | ATC | J06BA |
| Autoimmune disease | Rheumatoid arthritis | ICD-9/10-SE | M05, M06, 714 |
|  | Inflammatory bowel disease | ICD-9/10-SE | K50-K52, 555 |
|  | Multiple sclerosis | ICD-10-SE | G35 |
|  | Autoimmune hepatitis | ICD-10-SE | K754 |
|  | Systemic lupus erythematosus | ICD-9/10-SE | M32, 710A |
|  | Psoriatic arthritis | ICD-10-SE | L405, M073 |
|  | Ankylosing spondylitis | ICD-9/10-SE | M45, 720A |
|  | Giant cell arteritis | ICD-9/10-SE | M315, M316, 446F |
|  | Polymyalgia rheumatica | ICD-9/10-SE | M353, 725 |
|  | Sjögren/sicca syndrome | ICD-9/10-SE | M350, 710C |
|  | Systemic sclerosis | ICD-9/10-SE | M34, 710B |
| Diabetes | Diabetes | ICD-9/10-SE | E10, E11, 250 |
|  | Antidiabetics | ATC | A10 |
| Chronic obstructive pulmonary disease | Chronic obstructive pulmonary disease | ICD-9/10-SE | J20, J40-J44, 491, 492 |
| Asthma | Asthma | ICD-9/10-SE | J45, J46, 493 |
| Renal failure/chronic kidney disease | Renal failure/chronic kidney disease | ICD-9/10-SE | N17-N19, 584-586 |
| Glomerular disease | Glomerular disease | ICD-9/10-SE | N00-N08, 580, 582, 583 |
| Dementia/Alzheimer’s disease | Dementia/Alzheimer’s disease | ICD-9/10-SE | G30, F00-F02, F039, 290 |
|  | Anti-dementia drugs | ATC | N06D |
| Liver disease | Liver disease | ICD-10-SE | K70-K77 |
| Down syndrome | Down syndrome | ICD-10-SE | Q90 |
| Human immunodeficiency virus/acquired immune deficiency syndrome | Human immunodeficiency virus/acquired immune deficiency syndrome | ICD-9/10-SE | B20-B24, 279K |
| Sepsis | Sepsis | ICD-10-SE | A40, A41 |
| Influenza | Influenza | ICD-10-SE | J09-J11 |
| Pneumonia | Pneumonia | ICD-10-SE | J12-J18 |
| Solid organ transplantation | Heart | KVÅ | FQ |
|  | Lung | KVÅ | GDG |
|  | Small intestine | KVÅ | JFE |
|  | Liver | KVÅ | JJC |
|  | Pancreas | KVÅ | JLE |
|  | Kidney | KVÅ | KAS |
| Alcohol intoxication | Alcohol intoxication | ICD-9/10-SE | F10, 291, 303 |
| **Prescription medications** |  |  |  |
| Antithrombotics | Antithrombotics | ATC | B01A |
| Lipid-modifying agents | Lipid-modifying agents | ATC | C10A |
| Proton-pump inhibitors | Proton-pump inhibitors | ATC | A02BC |
| Corticosteroids, systemtic | Systemic corticosteroids | ATC | H02 |
| Immunosuppressants | Immunosuppressants | ATC | L04A |
| Antivirals | Antivirals | ATC | J05A |
| Opioids | Opioids | ATC | N02A |
| Abbreviations: ATC, Anatomical Therapeutic Chemical; ICD-7/9/10-SE, International Classification of Diseases, 7^th^/9^th^/10^th^ Revision, Swedish Version; KVÅ, *Klassifikation av vårdåtgärder* (Swedish classification system of medical and surgical procedures)  ^a^ICD-7-SE codes were obtained from the Swedish Cancer Registry. From this registry, diagnoses were included in the analysis if the tumor was recorded as a type that seldom or normally metastasizes or if the malignancy status was unknown or not yet determined. Benign or in situ tumors were excluded. | | | |

**Supplemental Table 2** Definitions of demographic variables

| **Variable** | **Values** | **Definition** | **Comment** | **Data Source** |
| --- | --- | --- | --- | --- |
| Sex | Woman |  |  | Statistics Sweden |
|  | Man |  |  |  |
| Age |  | Years from date of birth (YYYY-MM-15) to baseline | Only the year and month of birth were available, so we imputed the day of birth as the 15^th^. | Statistics Sweden |
| Birth in Sweden | Yes |  | Assumed “No” if country of birth was missing | Statistics Sweden |
|  | No |  |  |  |
| Highest level of completed education | Primary | SUN2000Niva_Old = 1, 2 | SUN2000Niva_Old is a Swedish classification system for highest level of completed education (*Utbildningsnivå, högsta, aggregerat till ”svenska” nivåer*)  Available only for persons who were aged ≥15 years on December 31, 2018. | Statistics Sweden (Longitudinal Integrated Database for Health Insurance and Labour Market Studies) |
|  | Secondary | SUN2000Niva_Old = 3, 4 |  |  |
|  | Post-secondary, <3 years | SUN2000Niva_Old = 5 |  |  |
|  | Post-secondary, ≥3 years | SUN2000Niva_Old = 6, 7 |  |  |
| Quintile of disposable family income in 2018 | 1-5 | We calculated quintiles based on the income distribution among the general-population controls.  Disposable family income in 2018 (*DispInkFam04 [Disponibel inkomst, familj, enligt 2004 års definition*]) was available in Swedish Krona. | Available only for persons who were aged ≥15 years on December 31, 2018 | Statistics Sweden (Longitudinal Integrated Database for Health Insurance and Labour Market Studies) |
| Residence in long-term care facility in 2019 or 2020 | Yes | BOFORM=2 (*boendeform = särskilt boende*) or SBO=1 (*säskilt boende = ja*) |  | National Board of Health and Welfare (Register for Care and Services for the Elderly and for Persons with Impairments According to the Social Services Act) |
|  | No | If not “Yes” |  |  |
| Use of homemaker service in 2019 or 2020 | Yes | HTJ = 1 (*Hemtjänst = ja*) |  | National Board of Health and Welfare (Register for Care and Services for the Elderly and for Persons with Impairments According to the Social Services Act) |
|  | No | If not “Yes” |  |  |
| Residence in Stockholm | Yes | Residence in Stockholm County on December 31, 2019  Boendekommun = 01XX |  | Statistics Sweden |
|  | No | Residence in other Swedish county on December 31, 2019  Boendekommun ≠ 01XX |  |  |


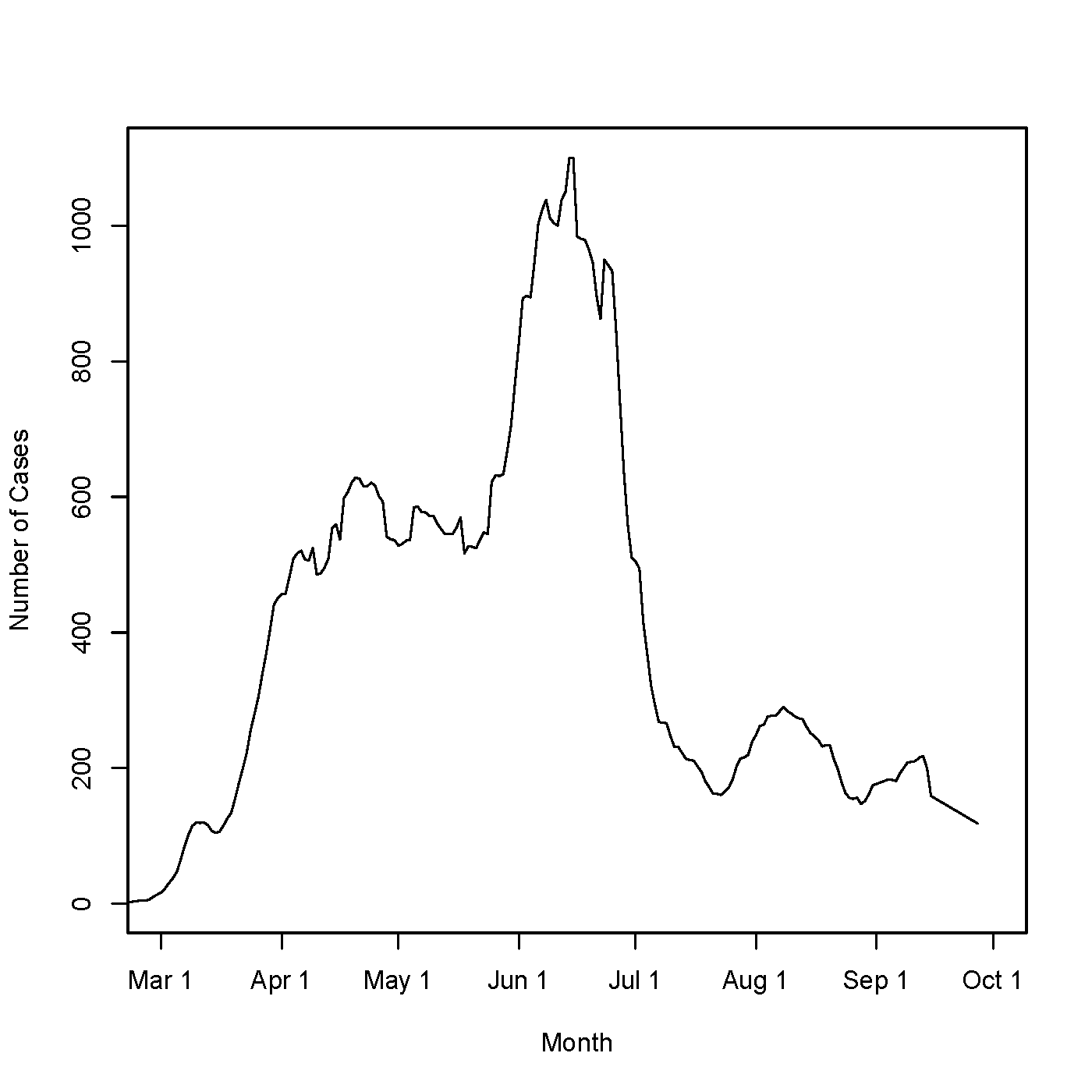


**Supplemental Fig. 1** Number of COVID-19 cases (7-day moving average) in Sweden from March to September 2020


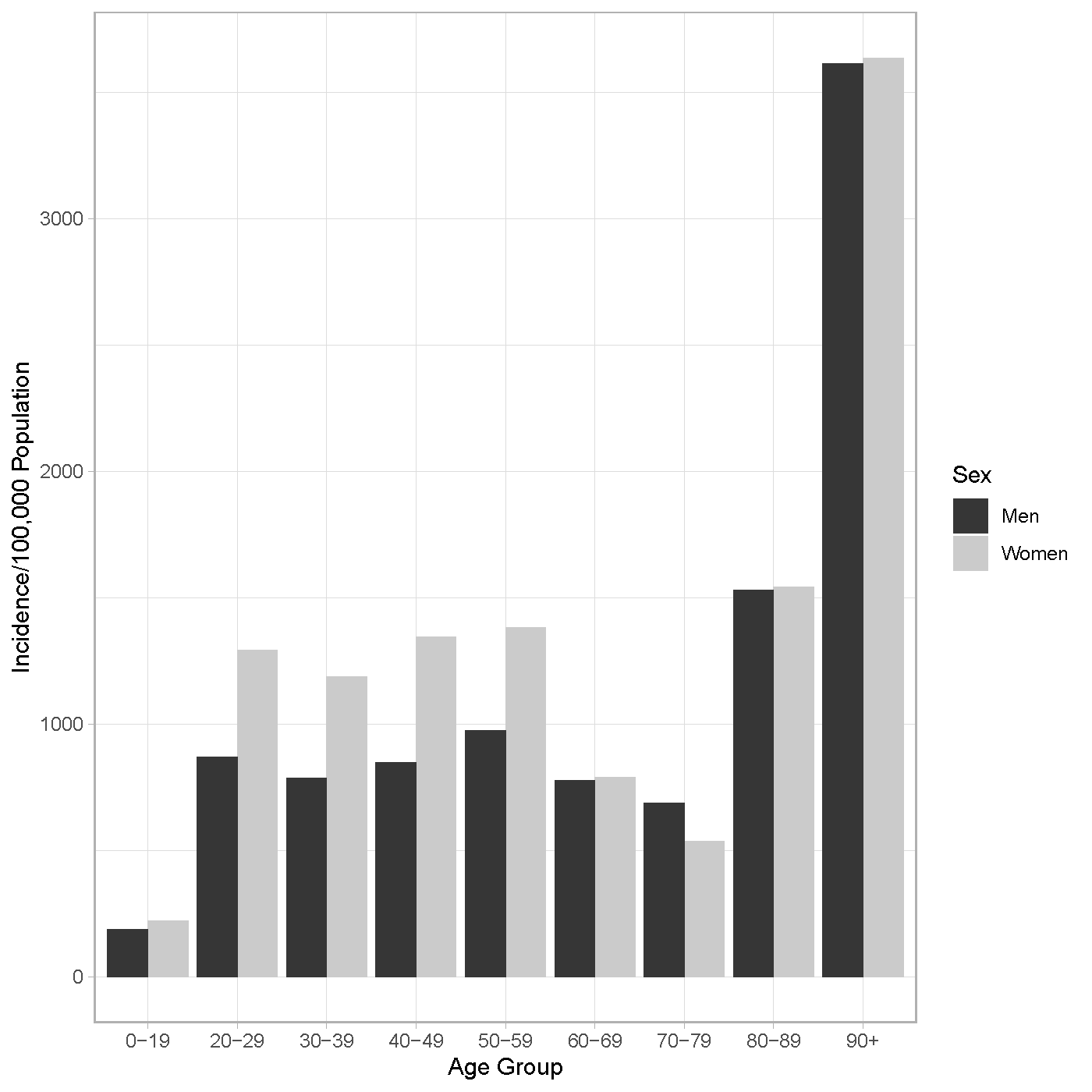


**Supplemental Fig. 2** Incidence of COVID-19 in Sweden until September 2020 by age group and sex

**Supplemental Table 3** Association between potential risk factors and COVID-19 (reference group: general population controls)

|  | **Adjusted^a^ Odds Ratio (95% CI)** | | |
| --- | --- | --- | --- |
| **Variable** | **Diagnosis** | **Non-ICU hosp.** | **ICU hosp.** |
| **Demographics** |  |  |  |
| Male sex | 0.65 (0.64-0.66) | 1.42 (1.37-1.47) | 2.75 (2.52-3.01) |
| Age group, y |  |  |  |
| 0-19 | 1 (ref) | 1 (ref) | 1 (ref) |
| 20-29 | 4.99 (4.82-5.17) | 3.75 (3.12-4.51) | 6.08 (3.91-9.48) |
| 30-39 | 4.16 (4.01-4.31) | 6.98 (5.89-8.28) | 6.34 (4.10-9.82) |
| 40-49 | 4.22 (4.07-4.37) | 11.56 (9.80-13.62) | 15.53 (10.29-23.44) |
| 50-59 | 4.04 (3.89-4.19) | 15.36 (13.06-18.07) | 31.20 (20.84-46.71) |
| 60-69 | 2.12 (2.03-2.21) | 13.31 (11.29-15.69) | 36.55 (24.37-54.81) |
| 70-79 | 1.16 (1.10-1.22) | 14.25 (12.07-16.82) | 24.11 (15.97-36.41) |
| 80-89 | 3.10 (2.94-3.28) | 29.17 (24.67-34.50) | 11.49 (7.33-18.00) |
| ≥90 | 8.68 (8.13-9.26) | 47.51 (39.77-56.76) | 2.13 (0.74-6.16) |
| **Comorbitities** |  |  |  |
| Cancer | 0.96 (0.92-0.99) | 1.02 (0.97-1.07) | 0.81 (0.71-0.92) |
| Hypertension | 1.03 (1.01-1.06) | 1.63 (1.56-1.71) | 1.85 (1.69-2.04) |
| Cardiovascular disease | 1.31 (1.27-1.36) | 1.66 (1.58-1.73) | 1.02 (0.91-1.15) |
| **Perscription medications** |  |  |  |
| Corticosteroids | 1.17 (1.15-1.19) | 1.47 (1.41-1.52) | 1.42 (1.30-1.55) |
| Opioids | 1.28 (1.26-1.30) | 1.70 (1.63-1.76) | 1.40 (1.29-1.53) |
| ^a^Adjusted for all variables in column 1 | | | |

**Supplemental Table 4** Association between potential risk factors and COVID-19 (reference group: general population controls)

|  | **Adjusted**^a^ **Odds Ratio (95% CI)** | | |
| --- | --- | --- | --- |
| **Variable** | **Diagnosis** | **Non-ICU hosp.** | **ICU hosp.** |
| **Demographics** |  |  |  |
| Male sex | 0.64 (0.63-0.65) | 1.30 (1.25-1.35) | 2.53 (2.31-2.77) |
| Age group, y |  |  |  |
| 0-19 | 1 (ref) | 1 (ref) | 1 (ref) |
| 20-29 | 5.28 (5.10-5.47) | 4.26 (3.54-5.12) | 6.52 (4.19-10.16) |
| 30-39 | 4.54 (4.38-4.70) | 8.51 (7.18-10.08) | 7.09 (4.58-10.96) |
| 40-49 | 4.70 (4.53-4.87) | 14.79 (12.56-17.42) | 17.84 (11.84-26.88) |
| 50-59 | 4.54 (4.38-4.71) | 20.09 (17.10-23.60) | 35.84 (23.99-53.55) |
| 60-69 | 2.38 (2.28-2.49) | 17.35 (14.74-20.43) | 40.77 (27.25-61.02) |
| 70-79 | 1.30 (1.23-1.36) | 18.48 (15.68-21.78) | 25.92 (17.21-39.04) |
| 80-89 | 3.52 (3.34-3.71) | 39.76 (33.69-46.92) | 12.74 (8.16-19.88) |
| ≥90 | 9.97 (9.36-10.63) | 69.20 (58.05-82.49) | 2.55 (0.88-7.36) |
| **Comorbitities** |  |  |  |
| Cardiovascular disease | 1.34 (1.29-1.39) | 1.68 (1.61-1.76) | 0.99 (0.89-1.12) |
| Hypertension | 1.06 (1.03-1.09) | 1.57 (1.50-1.65) | 1.64 (1.48-1.81) |
| Diabetes | 1.21 (1.17-1.25) | 2.09 (2.00-2.19) | 2.47 (2.24-2.73) |
| ^a^Adjusted for all variables in column 1 | | | |

**Supplemental Table 5** Association between potential risk factors and COVID-19 (reference group: general population controls)

|  | **Adjusted**^a^ **Odds Ratio (95% CI)** | | |
| --- | --- | --- | --- |
| **Variable** | **Diagnosis** | **Non-ICU hosp.** | **ICU hosp.** |
| **Demographics** |  |  |  |
| Male sex | 0.64 (0.63-0.65) | 1.30 (1.25-1.35) | 2.53 (2.31-2.77) |
| Age group, y |  |  |  |
| 0-19 | 1 (ref) | 1 (ref) | 1 (ref) |
| 20-29 | 5.28 (5.10-5.47) | 4.26 (3.54-5.13) | 6.53 (4.19-10.17) |
| 30-39 | 4.54 (4.38-4.70) | 8.51 (7.18-10.08) | 7.09 (4.59-10.97) |
| 40-49 | 4.70 (4.54-4.87) | 14.79 (12.56-17.42) | 17.84 (11.84-26.89) |
| 50-59 | 4.56 (4.40-4.73) | 20.10 (17.10-23.61) | 35.81 (23.97-53.52) |
| 60-69 | 2.41 (2.31-2.51) | 17.37 (14.76-20.46) | 40.70 (27.19-60.93) |
| 70-79 | 1.32 (1.25-1.39) | 18.52 (15.71-21.83) | 25.86 (17.16-38.98) |
| 80-89 | 3.58 (3.40-3.77) | 39.85 (33.75-47.04) | 12.71 (8.14-19.85) |
| ≥90 | 10.03 (9.41-10.70) | 69.26 (58.10-82.57) | 2.54 (0.88-7.36) |
| **Comorbitities** |  |  |  |
| Cardiovascular disease | 1.37 (1.32-1.42) | 1.69 (1.61-1.77) | 0.99 (0.88-1.12) |
| Hypertension | 1.08 (1.05-1.10) | 1.58 (1.50-1.66) | 1.63 (1.47-1.81) |
| Diabetes | 1.24 (1.19-1.28) | 2.10 (2.00-2.20) | 2.46 (2.21-2.73) |
| **Perscription medications** |  |  |  |
| Lipid-modifying agents | 0.92 (0.89-0.96) | 0.99 (0.94-1.04) | 1.02 (0.91-1.14) |
| ^a^Adjusted for all variables in column 1 | | | |

**Supplemental Table 6** Characteristics of COVID-19 cases and general-population controls aged 0-19 years

| **Variable** | **Controls**  **(n=100,221)** | **Diagnosis**  **(n=4,556)** | **Non-ICU hosp.**  **(n=161)** | **ICU hosp.**  **(n=25)** |
| --- | --- | --- | --- | --- |
| **Demographics** |  |  |  |  |
| Male sex, n (%) | 51,546 (51.4) | 2,134 (46.8) | 83 (51.9) | 11 (44.0) |
| Age, y |  |  |  |  |
| Mean (SD) | 10 (6) | 15 (5) | 9 (8) | 12 (7) |
| Median (IQR) | 10 (5-14) | 18 (14-19) | 10 (0-17) | 14 (5-17) |
| Born in Sweden, n (%) | 88,887 (88.7) | 4,002 (87.8) | 94 (58.4) | 20 (80.0) |
| Stockholm residence^a^ |  |  |  |  |
| Missing | 510 | 62 | 41 | 2 |
| N (%) | 23,935 (24.0) | 759 (16.9) | 39 (32.5) | 10 (43.5) |
| **Comorbitities, n (%)** |  |  |  |  |
| Any comorbidity/medication | 26,854 (26.8) | 1,904 (41.8) | 69 (42.9) | 10 (40.0) |
| Cardiovascular disease | 124 (0.1) | 11 (0.2) | 2 (1.2) | 0 (0.0) |
| Hypertension | 682 (0.7) | 71 (1.6) | 10 (6.2) | 3 (12.0) |
| Cancer | 210 (0.2) | 23 (0.5) | 9 (5.6) | 1 (4.0) |
| Immune disorder | 279 (0.3) | 17 (0.4) | 1 (0.6) | 0 (0.0) |
| Autoimmune disease | 1,849 (1.8) | 151 (3.3) | 5 (3.1) | 1 (4.0) |
| Diabetes | 483 (0.5) | 44 (1.0) | 6 (3.7) | 1 (4.0) |
| COPD | 4334 (4.3) | 197 (4.3) | 13 (8.1) | 0 (0.0) |
| Asthma | 10,797 (10.8) | 645 (14.2) | 25 (15.5) | 3 (12.0) |
| Renal failure/chronic kidney disease | 115 (0.1) | 13 (0.3) | 2 (1.2) | 1 (4.0) |
| Glomerular disease | 127 (0.1) | 11 (0.2) | 1 (0.6) | 0 (0.0) |
| Liver disease | 86 (0.1) | 4 (0.1) | 2 (1.2) | 0 (0.0) |
| Down syndrome | 124 (0.1) | 4 (0.1) | 2 (1.2) | 1 (4.0) |
| HIV/AIDS | 14 (0.0) | 1 (0.0) | 0 (0.0) | 0 (0.0) |
| Sepsis | 188 (0.2) | 16 (0.4) | 7 (4.3) | 1 (4.0) |
| Influenza | 482 (0.5) | 36 (0.8) | 8 (5.0) | 1 (4.0) |
| Pneumonia | 3442 (3.4) | 229 (5.0) | 16 (9.9) | 3 (12.0) |
| Solid organ transplantation | 16 (0.0) | 1 (0.0) | 1 (0.6) | 0 (0.0) |
| **Prescription medications, n (%)** |  |  |  |  |
| Antithrombotics | 343 (0.3) | 47 (1.0) | 5 (3.1) | 1 (4.0) |
| Proton-pump inhibitors | 4480 (4.5) | 521 (11.4) | 31 (19.3) | 2 (8.0) |
| Corticosteroids, systemic | 8352 (8.3) | 666 (14.6) | 27 (16.9) | 5 (20.0) |
| Immunosuppressants | 313 (0.3) | 50 (1.1) | 9 (5.6) | 1 (4.0) |
| Antivirals | 1,058 (1.1) | 143 (3.1) | 12 (7.5) | 1 (4.0) |
| Opioids | 2,032 (2.0) | 313 (6.9) | 10 (6.2) | 1 (4.0) |
| Abbreviations: COPD, chronic obstructive pulmonary disease; HIV/AIDS, human immunodeficiency virus/acquired immune immunodeficiency syndrome; hosp., hospitalization; ICU, intensive care unit; IQR, interquartile range; SD, standard deviation  ^a^On December 31, 2019 | | | | |

**Supplemental Table 7** All-cause mortality in COVID-19 cases and general-population controls aged 0-19 years

| **Variable** | **Control**  **(n=100,221)** | **Diagnosis**  **(n=4,555)** | **Non-ICU hosp.**  **(n=161)** | **ICU hosp.**  **(n=25)** |
| --- | --- | --- | --- | --- |
| Number of deaths |  |  |  |  |
| 30 days | 1 | 2 | 1 | 0 |
| 60 days | 3 | 2 | 1 | 1 |
| 90 days | 3 | 2 | 1 | 1 |
| Total | 4 | 2 | 1 | 1 |
| Person-months at risk | 401,728 | 13,981 | 746 | 97 |
| Mortality rate/1,000 person-months | 0.01 | 0.1 | 1.3 | 10.3 |
| Mortality, % (95% CI) |  |  |  |  |
| 30 days | 0.0 (0.0-0.0) | 0.0 (0.0-0.1) | 0.6 (0.0-1.8) | 0.0 (0.0-0.0) |
| 60 days | 0.0 (0.0-0.0) | 0.0 (0.0-0.1) | 0.6 (0.0-1.8) | 4.3 (0.0-12.3) |
| 90 days | 0.0 (0.0-0.0) | 0.0 (0.0-0.1) | 0.6 (0.0-1.8) | 4.3 (0.0-12.3) |
| Abbreviations: hosp., hospitalization; ICU, intensive care unit | | | | |
